# Transcriptome Profiling Reveals Differential Gene Expression of Secreted Proteases and Highly Specific Gene Repertoires Involved in *Lactarius–Pinus* Symbioses

**DOI:** 10.3389/fpls.2021.714393

**Published:** 2021-08-19

**Authors:** Nianwu Tang, Annie Lebreton, Wenjun Xu, Yucheng Dai, Fuqiang Yu, Francis M. Martin

**Affiliations:** ^1^Germplasm Bank of Wild Species, Yunnan Key Laboratory for Fungal Diversity and Green Development, Kunming Institute of Botany, Chinese Academy of Sciences, Kunming, China; ^2^Beijing Advanced Innovation Center for Tree Breeding by Molecular Design, Beijing Forestry University, Beijing, China; ^3^Centre INRAE-GrandEst Nancy, INRAE, UMR Interactions Arbres/Microorganismes, Université de Lorraine, Champenoux, France

**Keywords:** genomics, evolution, milkcap mushrooms, proteases, host-specificity

## Abstract

Ectomycorrhizal fungi establish a mutualistic symbiosis in roots of most woody plants. The molecular underpinning of ectomycorrhizal development was only explored in a few lineages. Here, we characterized the symbiotic transcriptomes of several milkcap species (*Lactarius*, Russulales) in association with different pine hosts. A time-course study of changes in gene expression during the development of *L. deliciosus–Pinus taeda* symbiosis identified 6 to 594 differentially expressed fungal genes at various developmental stages. Up- or down-regulated genes are involved in signaling pathways, nutrient transport, cell wall modifications, and plant defenses. A high number of genes coding for secreted proteases, especially sedolisins, were induced during root colonization. In contrast, only a few genes encoding mycorrhiza-induced small secreted proteins were identified. This feature was confirmed in several other *Lactarius* species in association with various pines. Further comparison among all these species revealed that each *Lactarius* species encodes a highly specific symbiotic gene repertoire, a feature possibly related to their host-specificity. This study provides insights on the genetic basis of symbiosis in an ectomycorrhizal order, the Russulales, which was not investigated so far.

## Introduction

Ectomycorrhiza (EcM) is a mutualistic association formed between soil-borne ectomycorrhizal fungi and roots of most woody plants. In this symbiosis, the fungus assists the host plant in mineral nutrients and water uptake, while the host plant transfers glucose to the fungus (Smith and Read, 2008). As most dominant tree species, such as pines, oaks, and poplars, in temperate and boreal forests are able to form EcM with various EcM fungi, this symbiosis may have a profound influence on the functioning and stabilization of forest ecosystems (Brundrett and Tedersoo, 2018; Tedersoo et al., 2020).

The establishment of EcM symbiosis entails a series of developmental stages, initiating with a molecular dialogue between rhizospheric hyphae and root cells, proceeding with hyphal proliferation on the root epidermis and culminating with the formation of characteristic extraradical mantle and intraradical Hartig net (Garcia et al., 2015; Genre et al., 2020). These steps require coordinated gene regulations in both partners. Transcriptomic studies have identified a large number of fungal or plant genes which are regulated during the symbiosis (Voiblet et al., 2001; Heller et al., 2008; Martin et al., 2008, 2010; da Silva Coelho et al., 2010; Doré et al., 2015; Kohler et al., 2015; Plett et al., 2015; Peter et al., 2016). Up- or down-regulation of genes related to signaling pathways, nutrient transport, cell wall modifications, and plant defenses have been characterized in most EcM interactions investigated so far, indicating their importance for the symbiosis development. In contrast, the up-regulation of genes coding for effector-like mycorrhiza-induced small secreted proteins, so-called MiSSPs, is mostly species-specific. For example, the induction of the *LbMiSSP7* gene in *Laccaria bicolor* is required for the colonization of *Populus* roots. By interacting with the JASMONATE ZIM DOMAIN (JAZ6) transcription factor, the secreted symbiotic effector MiSSP7 is dampening the host defense pathways (Plett et al., 2011, 2014; Daguerre et al., 2020). Additional MiSSPs have been characterized in *L. bicolor* and *Pisolithus microcarpus* (Pellegrin et al., 2019; Kang et al., 2020; Plett et al., 2020). Recent large-scale analyses confirmed the lineage-specificity of MiSSPs (Kohler et al., 2015; Miyauchi et al., 2020) and it has been suggested that these specific genes may play a role in EcM host-specificity (Liao et al., 2016; Martin et al., 2016).

Comparative transcriptomic analyses have shown that EcM fungi, such as *P. microcarpus*, *L. bicolor* or *Suillus* spp., induced different sets of genes when colonizing different hosts (Plett et al., 2015; Liao et al., 2016). How host specificity may be encoded into EcM fungal genomes and driven by gene expression remains poorly understood. In their global analysis of gene family dynamics for 23 *Suillus* species, Lofgren et al. (2021) found that host specialist *Suillus* genomes encode numerous rapidly evolving gene families, representing both gene family expansions and contractions. Gene families coding for terpene- and non-ribosomal peptide synthetase-like secondary metabolite clusters, known to play a role in the structuring of host specificity relationships for fungi, are significantly enriched in *Suillus* species. In contrast, they found no evidence of significant differences in the abundance of small secreted proteins (SSPs), or G-protein coupled receptors between *Suillus* and other EcM fungi. Additionally, the comparisons among *Suillus* species specializing on different host groups identified no genomic differences among the three classes of genes investigated, with the possible exception of SSPs, which showed a loss of richness in red pine-associated species relative to larch-associated species.

Milkcap fungi (*Lactarius* spp., Russulaceae) are an EcM lineage distributed in many temperate forests (Verbeken and Nuytinck, 2013). This lineage is distantly related to and independent from the mostly studied models in Agricales and Boletales (Miyauchi et al., 2020). This situation makes its comparison with other lineages of great importance for understanding the commonality and specificity of symbiotic gene repertoires. Moreover, it has long been documented as host specialists (Looney et al., 2018), thus representing a suitable model for the study of EcM host-specificity. Within the framework of the Mycorrhizal Genomics Initiative (Kohler et al., 2015; Miyauchi et al., 2020), we have sequenced the genome of several *Lactarius* species in the section of Deliciosi to identify the genomic imprint pertaining to host-specificity (Lebreton et al., in preparation). The aim of the present study was to identify the fungal gene sets involved in *Lactarius–Pinus* ectomycorrhizal interactions, with a special focus on the genes possibly involved in the host-specificity. To this end, the symbiotic transcriptome of four *Lactarius* species (*L. deliciosus, L. akahatsu*, *L. sanguifluus*, and *L. vividus*), were sequenced and compared to their free-living mycelial control. Symbiosis-regulated genes in each interaction were identified, and then compared to uncover the genetic commonality and specificity of *Lactarius* symbioses. To better understand the gene expression dynamics underlying *Lactarius* EcM development, a time-course transcriptomics was performed on the interaction between *L. deliciosus* and *Pinus taeda*. This study identified several features in the *Lactarius* symbiosis-related gene repertoires which have not been reported in other EcM lineages so far, thus providing new insights on the molecular mechanisms underlying the development of ectomycorrhizal symbioses.

## Materials and Methods

### Mycorrhiza Formation Using *Lactarius* Species

Dikaryotic vegetative mycelia of *L. deliciosus, L. akahatsu*, *L. sanguifluus*, and *L. vividus* were used for the EcM formation. To find out the compatible host species for each *Lactarius*, an assay involving five pine species (*P. massoniana*, *P. sylvestris*, *P. taeda*, *P. tabuliformis*, and *P. yunnanensis*) was performed mainly using an *in vitro* “pouch” coculture system modified from Guerin-Laguette et al.(2000; Supplementary Figure 1). The combinations involving *P. tabuliformis* and *P. yunnanensis* were assayed with a traditional pot system described in Wang et al. (2019), since aseptic seedlings could not be produced. Pine seeds were sterilized with 10% H_2_O_2_ solution and germinated on 1% agar plate at 25°C, 14/10 h (day/night). Germinated seeds were then transferred to test tubes (containing perlite/vermiculite mix) for further growth until enough lateral roots developed (ca. 8–10 weeks). Fast-growing mycelia was prepared 2 weeks before coculture by sub-culturing mycelial agar plugs (ca. 0.5 cm × 0.5 cm) on fresh ½ MMN + ½ PDA medium (Wang et al., 2019), covered with a cellophane membrane.

For coculturing, a membrane with mycelial plugs was placed onto the filter paper to interact with pine roots. A low-concentration MES buffer (1.25 mM, PH 5.6) described in Guerin-Laguette et al. (2000), was used to promote the mycorrhization. Cultures were arranged vertically, and the lower part of the pouch was covered with an aluminum foil bag to prevent light from reaching the fungus and roots. For *L. akahatsu*, *L. sanguifluus*, and *L. vividus*, the mature ectomycorrhizal root tips/clusters formed on *P. tabuliformis*, *P. sylvestris*, and *P. taeda*, respectively, were harvested for microscopy and RNA extraction. In *L. deliciosus–P. taeda* interaction, materials of 2, 4, and 6 weeks post inoculation (wpi), corresponding to early, middle, and late stage of EcM development based on morphological and anatomical features, were sampled. At 2 wpi, we collected both young ectomycorrhizal root tips (primordia) and mycelia and rootlets in the vicinity. Only the ectomycorrhizal clusters were collected at 4 and 6 wpi stages. Free-living mycelium control was prepared on the same medium covered with a cellophane for 2 weeks. Both mycorrhizal and control mycelial samples were prepared with five replicates (i.e., five pouches or pots).

### Microscopic Observation

To determine the extent of root colonization by *Lactarius* mycelium and formation of ectomycorrhizal root tips, pine seedlings were observed weekly using a Leica M205C stereomicroscope. Samples were taken for sectioning at each time point. To check the formation of the intraradical Hartig net, we subjected representative root tips or ectomycorrhizal clusters to propidium iodide (PI)/wheat germ agglutinin (WGA) double staining for plant and fungal structures, respectively. Root tips or clusters were fixed overnight in 4% paraformaldehyde solution at 4°C. After a dehydration using a series of ethanols (15, 30, 50, 70, 90, 95, and 100%), they were cleared with Histo-clear II (HS-202, National Diagnostics) and then embedded in pure paraffin wax for sectioning. 5-μm transverse sections were prepared using a Microtome (HM340E, Thermo Scientific). After a dewaxing and rehydration, sections were stained in 10 μg/ml PI (25535-16-4, Sigma-Aldrich, diluted in PBS), for 5 min, and then counterstained overnight in 5 μg/ml WGA conjugated with FITC (WGA-FITC, L4895, Sigma-Aldrich, diluted in PBS) at 4°C. After washing with PBS, all sections were observed with a Leica TCS SP8 confocal scanning laser microscope equipped with a HCX PL APO 40x/0.85 objective. WGA-FITC and PI were excited at the 488 nm wavelength of an argon laser. Fluorescence emission was detected at 493–534 nm for WGA-FITC and 622–702 nm for PI. Settings (laser intensity, gain, offset, and magnification) for observations were maintained consistently among all samples.

### RNA Extraction, cDNA Synthesis, and RNA Sequencing

Transcriptomes of the ectomycorrhizas and mycelial controls were sequenced at the Beijing Genomics Institute (BGI). All samples were sequenced in triplicate. Total RNAs from ectomycorrhizal root tips/clusters were isolated using a CTAB-LiCl method modified from Liao et al. (2014), whereas those from the control free-living mycelia were isolated using a plant RNA extraction kit (Tiangen, Cat.No DP441, Beijing, China) with genomic DNA removal step as per the manufacturer’s instructions. After quality control (RIN > 8.0) on an Agilent 2100 Bioanalyzer, qualifying RNA samples underwent mRNA isolation and sequencing library construction following the standard BGI SEQ protocol (Natarajan et al., 2019). RNA sequencing (RNAseq) was performed on the BGISEQ-500 platform with a 150 bp pair-end sequencing. FastQC was run for quality control. Adapter trimming and low-quality reads filtering were performed by BGI using custom scripts. All clean reads were submitted to National Center for Biotechnology Information Sequence Read Archive under the BioProject of PRJNA706172. Detailed RNAseq sample, library and sequencing information was summarized in Supplementary Table 1.

### Updating the *Lactarius* Gene Catalogs

RNA mapping to the *Lactarius* genome assemblies available at the MycoCosm database^1^ identified new genes not annotated by the JGI gene prediction pipeline. The existing gene annotations were thus updated as follows. Clean pair-end RNAseq reads from each library were mapped onto corresponding genomes using STAR v2.7.2b (1% mismatch allowed, max. intron size = 1,000 bp, reads mapping at >5 sites discarded, Dobin and Gingeras, 2015). Based on this mapping, *de novo* gene prediction was performed with StringTie v.2.0.3 (Min. size 300 bp, Pertea et al., 2015). *De novo* predicted genes not overlaping with pre-existing JGI gene models were added to the final gene catalog for subsequent analysis. We added 1,945, 2,222, 3,350, and 1,829 new gene models to JGI gene catalogs for *L. deliciosus, L. akahatsu, L. sanguifluus*, and *L. vividus* (Supplementary Table 2). Then, putative coding sequences were identified using TransDecoder v5.5.0 (Min. size 100 aa^2^). PFAM domains, lipases, proteases, and secreted proteins were predicted following our custom bioinformatic pipelines.

### Gene Expression Analysis

Filtered RNAseq reads were mapped onto their corresponding annotated *Lactarius* genomes as described above. Differentially expressed genes (DEGs) were identified using DEseq2 v1.28.1 (Love et al., 2014) by comparing normalized gene expression levels (i.e., Transcripts Per Million, TPM) in transcriptomes from ectomycorrhizal and free-living mycelia for each *Lactarius* species. Genes with a log_2_(fold-change; LFC) > 2 or < 2, and adjusted *p*-value < 0.05 were deemed to be differentially expressed. Figures displaying the gene expression ratio and gene clustering were performed using the R packages *pheatmap*, *ggtree*, and *ape*. The phylogenomic tree of the Russulales order was retrieved from Lebreton et al. (in preparation), whereas the phylogenetic tree of the 160 fungal species was retrieved from the aforementioned MycoCosm database.

## Results

### *In vitro* Formation of *Lactarius* EcM Symbioses

In order to produce symbiotic EcM samples for transcriptomic sequencing, a mycorrhiza formation assay was initially performed by using the four *Lactarius* species. After a 12-week coculture with five different pine species, each *Lactarius* species colonized only one or two *Pinus* species under our experimental conditions, confirming the high host-specificity of *Lactarius* species (Supplementary Figure 2A). We also noticed that a host preference, in term of mycorrhiza formation efficiency, might occur in the species colonizing two pines (data not shown). Based on this assay, we sampled the EcM root tips/clusters of each *Lactarius* species, formed on their most compatible hosts, for the RNAseq (Supplementary Figure 2A). These included *L. akahatsu* in association with *P. tabuliformis*, *L. sanguifluus* with *P. sylvestris*, *L. deliciosus*, and *L. vividus* with *P. taeda* (Supplementary Figures 2B–D). Microscopic observation confirmed the “mature” mycorrhizal status of these interactions (Supplementary Figure 2E). As *L. deliciosus*–*P. taeda* interaction displayed the highest efficiency (i.e., forming abundant EcM within the shortest time period), it was further chosen as a “model” for a time-course experiment.

### Development of *L. deliciosus–P. taeda* Ectomycorrhiza

**FIGURE 1:**
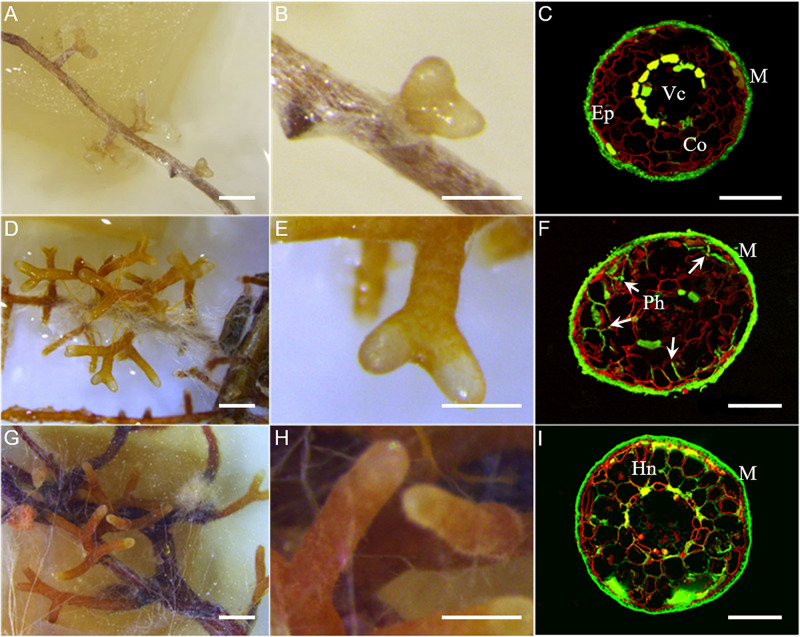
Time-course of *L. deliciosus*–*P. taeda* ectomycorrhizal roots. **(A–C)** 2 wpi, early developmental stage; **(D–F)** 4 wpi, middle developmental stage; and **(G–I)** 6 wpi, late developmental stage. Paraffin-embedded sections were stained with propidium iodide (red color, root cell walls) and WGA-FITC (green color, fungal hyphae). Abbreviations: Ep, epidermis; Co, cortex; Vc, vascular cylinder; M, fungal mantle; and Ph, penetrating hyphae (arrowheads) forming the Hartig net (Hn). Scale bar in **(A,D,G)** = 1 mm, in **(B,E,H)** = 500 μm, and in **(C,F,I)** = 100 μm.

The time-course of the development of *L. deliciosus–P. taeda* EcM was characterized by observing root development and dual fluorescence-stained transverse root sections. Two fluorescent markers were used, WGA-FITC for detection of fungal cell walls (green in Figure 1) and PI for visualization of plant cell walls (red in Figure 1). At 2 wpi, we observed discontinuous mycelial aggregates onto *P. taeda* roots and only a few EcM tips (Figures 1A,B). At this early stage of development, confocal microscopic observations showed the presence of a thin layer of hyphal mantle surrounding the roots, but no internal hyphal penetration (Figure 1C). At 4 wpi, highly branched clusters of ectomycorrhizal root tips were formed (Figures 1D,E). *L*. *deliciosus* had formed a full mantle around the lateral roots and had begun to grow into the intercellular space of the root (Figure 1F). Typically, at this middle stage of development, fungal hyphae had surrounded the rhizodermal cells of the root and reached the outer cortex (i.e., Hartig net). At 6 wpi, dense short extraradical mycelia, emanating from the mantle, resulted in a rougher surface on the mycorrhizal root clusters (Figures 1G,H). The fungus continued extending within the intercellular space of the root to surround one to two layers of cortical cells (Figure 1I). Based on these morphological stages observed repeatedly over the course of multiple experiments, we defined the 2 wpi time point as the early “aggregation” stage, the 4 wpi time point as the Hartig net “proliferation” stage, and the final, “mature” mycorrhizal root tip stage at 6 wpi.

### Gene Expression During the EcM Development

**FIGURE 2:**
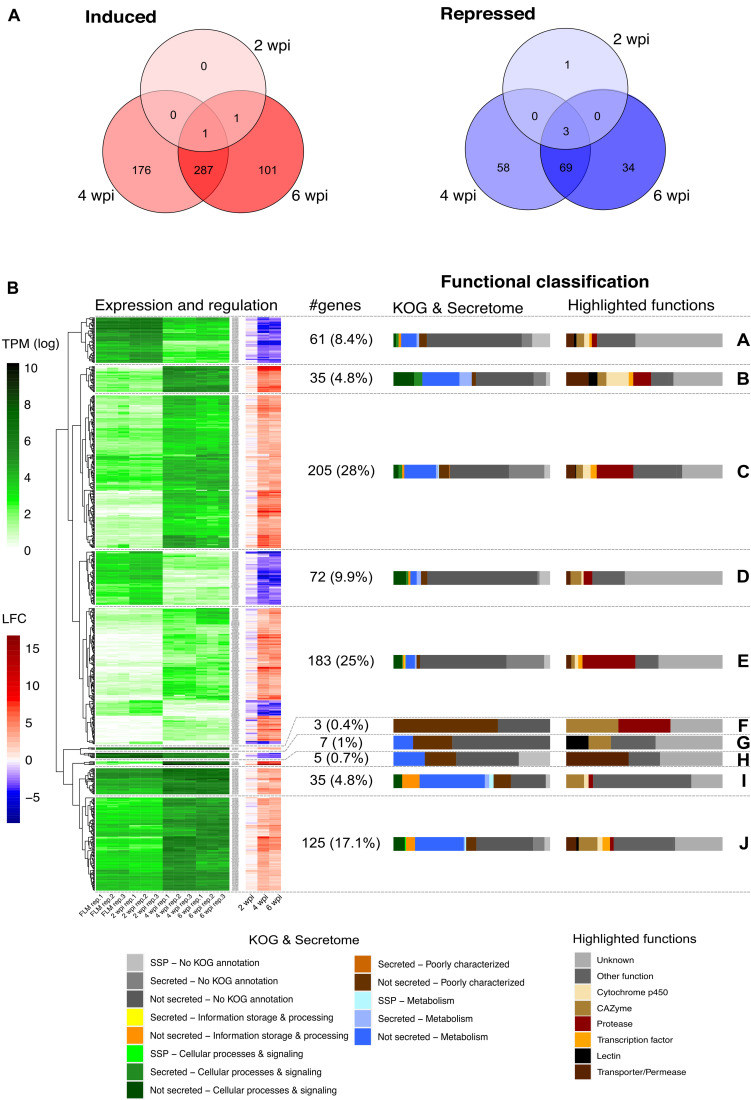
*L. deliciosus* differentially expressed genes (DEGs) at various developmental stages. **(A)** Venn diagram showing the overlap in DEG number at 2, 4, and 6 weeks post inoculation (wpi), as compared with the free-living mycelium (FLM); **(B)** Expression (TPM) and regulation (LFC) of the 731 DEG at 2, 4, and 6 wpi, as compared to the free-living mycelium (FLM). A complete linkage clustering of gene expression [log_2_(TPM)] allowed to identify 10 clusters (A to J). The regulation of the corresponding genes is displayed as a heatmap next to the expression. The functional classification of each cluster is shown as a bar plot on the right side.

To identify which gene sets are induced in the mycelium during the development of *L. deliciosus*–*P. taeda* symbiosis, RNA-seq transcript profiles at 2, 4, and 6 wpi were produced. Of the 20,138 genes of *L. deliciosus* (i.e., 18,193 genes annotated by JGI pipleline plus 1,945 newly predicted genes), 15,609 (77.5%, TPM > 1) were expressed in the free-living mycelium. In the mycelium colonizing pine roots, 2, 464, and 390 genes showed an increased transcript abundance, while 4, 130, and 106 displayed a reduced transcript abundance at 2, 4, and 6 wpi, respectively, (Figure 2A and Supplementary Tables 3, 4). The DEG set was higher at 4 wpi, indicating that the fungus underwent the greater amplitude of transcriptional reprogramming when initiating the root apoplast colonization. In contrast, there were only six DEG at the earlier developmental stage (2 wpi), even though several EcM root tips were already observed. A large DEG proportion (62–74% of up-regulated genes and 55–68% of down-regulated genes, Figure 2A) were shared between the middle (4 wpi) and late (6 wpi) developmental stages though they display very different Hartig net development.

Hierarchical clustering of the log_2_-transformed data of *L. deliciosus* DEGs in contact with pine roots for different lengths of time identified several sets of genes with coordinated differential expression, referred to as clusters (Figure 2B). The set of DEGs exhibiting the highest increase in transcript abundance was characterized by a high percentage of genes coding for proteases (clusters B, C, and E). DEGs with a low to moderate up-regulation are involved in core metabolism and signaling pathways (clusters C, I, and J and Supplementary Tables 3, 4). Besides, many genes coding for membrane transporters/permeases, carbohydrate-active enzymes (CAZymes), transcription factors and cytochrome P450 were also identified. The group of genes exhibiting decreased transcript abundance (clusters A, D, G, and some members of cluster E and Supplementary Tables 3, 4) was characterized by a high percentage of poorly characterized genes or genes of unknown function, although a few were annotated as CAZymes, membrane transporters/permeases, proteases, or cytochromes P450.

In the early developmental stage, only two genes were found to be significantly up-regulated, a major facilitator superfamily carboxylic acid transporter gene (Lacdel1|2527233) and a gene with an unknown function (Lacdel1|2532877). Notably, the former gene was also up-regulated at 4 and 6 wpi. The down-regulated genes code for secreted ceratoplatanin proteins (CPPs; Lacdel1|1784454, Lacdel1|1060664), secreted triacylglycerol lipase (TGL; Lacdel1|2672458), and a protein of unknown function and were repressed throughout EcM development.

At 4 wpi, 94 protease-coding genes were identified among the 464 up-regulated DEGs (Supplementary Tables 3, 4), whereas only seven protease genes were down-regulated. There were 35 up-regulated DEGs coding for CAZymes, including glycoside hydrolases, glycosyltransferases, and several other families. Induced secreted CAZymes target both fungal cell wall and plant cell wall polysaccharides (Supplementary Tables 3, 4). However, no polysaccharide lyase gene and gene families targeting pectin were found in these DEGs. Several DEGs coding for membrane transporters/permeases were identified, including those involved in oligopeptide, amino acid, sugar, carboxylic acid, nucleoside, water, and iron transport. The formation of the Hartig net also triggered the induction of genes coding for cytochrome P450, transcription factor, TGL, and lectins. A large portion of DEGs was found to have no homologues in other fungal genomes. In several ectomycorrhizal interactions, effector-like MiSSPs play a very important role, as these proteins are responsible for communicating between the two partners as well as dampening the host defense reactions (Martin et al., 2016). We only found 22 MiSSP genes and 12 mycorrhiza-repressed small secreted protein (MrSSP) genes in *L. deliciosus*. Of note, two of them encode GH45 potentially targeting cellulose in plant cell wall, one code for a M36 fungalysin and one for a TGL, while two CPP belong to MrSSPs.

At the final, “mature” mycorrhizal root tip stage (6 wpi), we found a lower number of up-regulated transcripts coding for proteases (76), CAZymes (20), cytochrome P450s (13), and SSPs (16), compared with 4 wpi (Supplementary Figure 3). Although a comparable number of transcription factor genes (15) were up-regulated, only half of them were shared with 4 wpi stage, suggesting substantial differences in regulation pathways. In contrast, most transporter/permease genes induced at 4 wpi were still up-regulated at 6 wpi.

### Regulation of Fungal Protease Genes During Symbiosis

**FIGURE 3:**
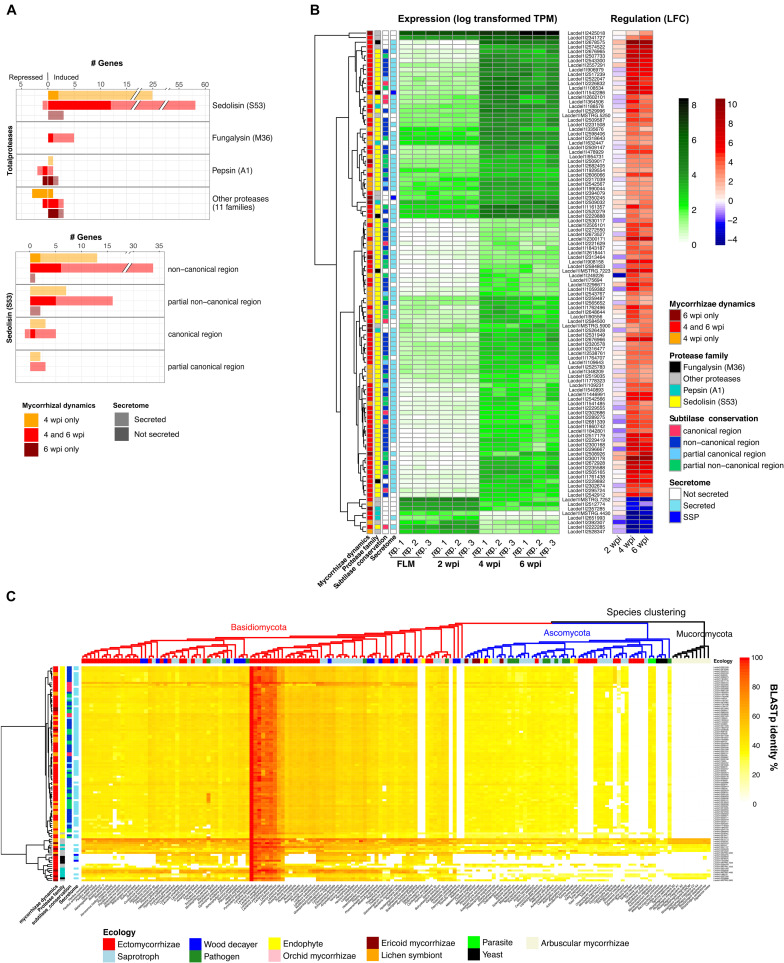
*L. deliciosus* differentially expressed genes (DEGs) coding for proteases. **(A)** Gene family classification of the 125 differentially expressed proteases and distribution of the conserved region in the sedolisin (S53) family; **(B)** Expression (TPM) and regulation (LFC) of differentially-expressed protease genes at 2, 4, and 6 wpi, as compared to the free-living mycelium (FLM); and **(C)** Evolutionary conservation of these proteases across 160 fungal species of various lineages and ecology (MycoCosm), as indicated by BLASTp identities.

As stressed above, *L. deliciosus* up-regulated DEGs are enriched (*p* < 10^–5^, Fisher exact test) in protease-coding genes (103 members), including 81 encoding secreted proteases. The most abundant induced proteases belong to the sedolisin (S53) family with 86 members, then followed by fungalysin (M36; Figure 3A). Most of the sedolisins (S53) contain a non-canonical domain. They were only induced at 4 and 6 wpi when the mycelium colonized the root apoplastic space (Figure 3B). Since induction of fungal sedolisin genes was not reported in other EcM symbioses (Kohler et al., 2015; Miyauchi et al., 2020), we assessed whether their protein sequences were conserved in other fungal species. A BLASTp query against 160 representative fungal proteomes retrieved from JGI Mycocosm database showed that most of *L. deliciosus* sedolisins are conserved in Dikarya (Figure 3C). We also noticed that four fungalysin genes induced in mycorrhiza could not be detected in most EcM fungi studied so far, but conserved in most basidiomycotan saprotrophs and pathogens (Figure 3C).

### Commonly and Specifically Regulated Genes in *Lactarius* Species

**FIGURE 4:**
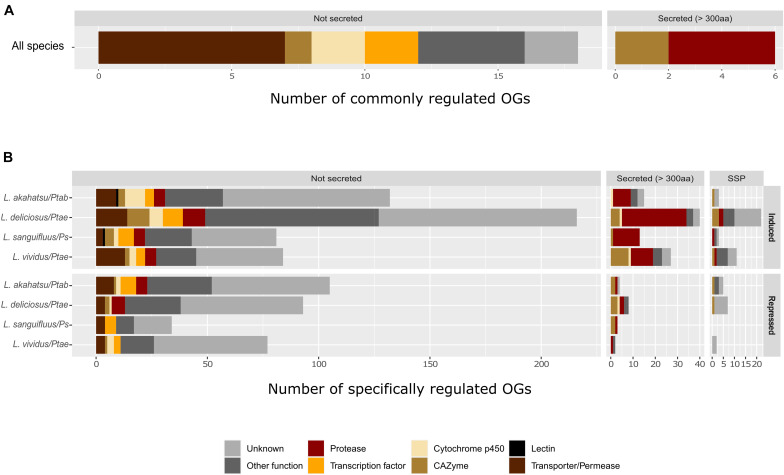
Functional classification of commonly **(A)** and specifically **(B)** regulated orthogroups (OGs) in *Lactarius* species. Only commonly induced OGs in all four fungal species were shown, since no commonly repressed OGs were found. Abbreviations: Ps, *P. sylvestris*; Ptab, *P. tabuliformis*; and Ptae, *P. taeda*.

Further transcriptomic profiling identified 321, 301, and 283 up-regulated genes, and 139, 73, and 117 down-regulated genes in *L. akahatsu*, *L. sanguifluus*, and *L. vividus*, respectively, during their symbioses. Comparison among four *Lactarius* species, based on protein orthology, identified only 24 orthogroups (OGs) containing DEGs induced in all species, and no such OG was found in down-regulated genes (Supplementary Figure 4 and Supplementary Tables 3, 4). We also noticed that *L. deliciosus* does not share more common OGs with *L. vividus* than with other two species, even though they were in association with the same pine host (*P. taeda*). Most of the commonly induced OGs code for proteins with known function: seven encode membrane transporters/permeases, including carboxylic acid, monosaccharide, and water transporters; four code for secreted proteases, including fungalysins (M36), pepsin (A1), and sedolisin (S53), and three encode plant cell wall degrading enzymes (GH3, GH47; Figure 4A). Zn-finger transcription factors and cytochrome P450s were also present in the annotation of this core set of DEGs (Supplementary Tables 3, 4).

Most of the OGs contained genes whose regulation was specific to a single *Lactarius* species. We identified 150, 278, 97, and 122 species-specfic up-regulated OGs and 114, 108, 37, and 81 species-specific down-regulated OGs in *L. akahatsu*, *L. deliciosus, L. sanguifluus*, and *L. vividus*, respectively, (Supplementary Figure 4). As shown in Figure 4B, the most abundant set of DEGs code for proteases, followed by membrane transporters/permeases, transcription factors, CAZymes, and cytochrome P450, a pattern similar for each individual *Lactarius* species (Supplementary Tables 3, 4). Of note, we identified MiSSP and lectin genes only in the specifically regulated OGs.

### Sequence Conservation of *Lactarius* DEGs in Russulales

**FIGURE 5:**
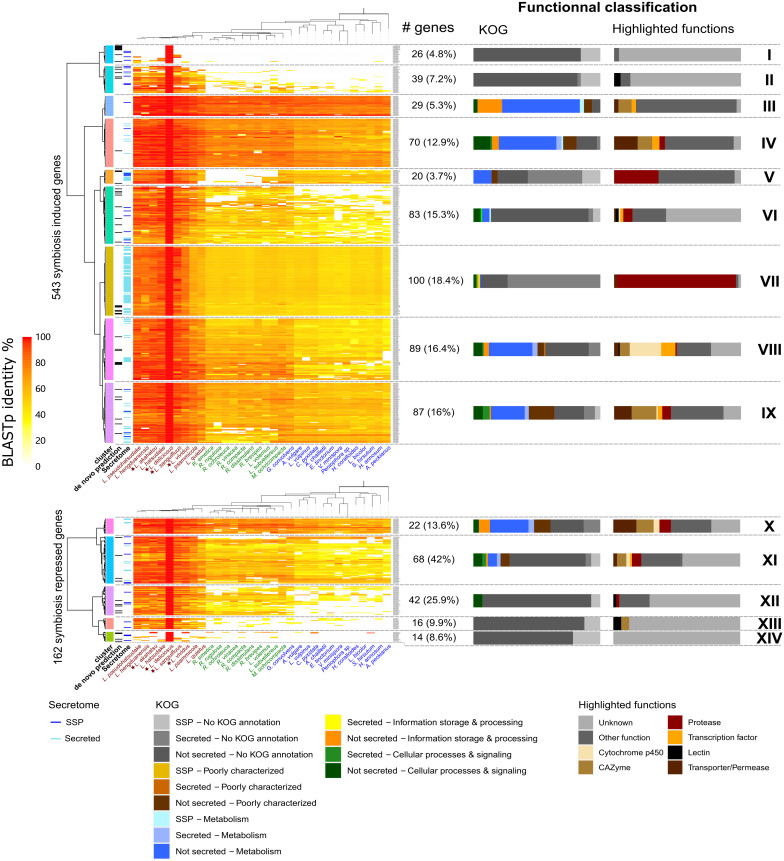
Phylogenetic conservation of *L. deliciosus* differentially expressed genes (DEGs). Heatmaps show BLASTp sequence identity of proteins encoded by *L. deliciosus* DEGs among 31 protein repertoires of Russulales. Functional categories (KOG and highlighted functions) are given for each cluster as barplots on the right panel. Data were visualized and clustered using R (package pheatmap, Euclidian distance, and Ward clustering method). As a few DEGs were annotated as non-coding, the number of symbiosis induced and repressed genes indicated here is slightly different from the Figure 2.

To assess the evolutionary conservation of *Lactarius* DEGs, we compared their protein sequences to the protein repertoire of 28 sequenced Russulales species at JGI^3^. Most *Lactarius* DEG-encoded proteins showed a high sequence similarity with proteins of the other Russulales species, including saprotrophic species, suggesting that most of *Lactarius* symbiosis-related genes were co-opted/recruited from their saprotrophic ancestors (Figure 5 and Supplementary Figures 5–7). The most conserved genes (clusters III, IV, X in *L. deliciosus*, III, IV, X in *L. akahatsu*, III, VIII in *L. sanguifluus*, and III, VII in *L. vividus*) code for proteins involved in primary metabolism, and information storage and processing pathways. On the other hand, several sets of *Lactarius*-specific DEGs have no homolog in other Russulales species (clusters I, II, XIII, XIV in *L. deliciosus*, I in *L. akahatsu*, I, X in *L. sanguifluus*, and I, IX, X in *L. vividus*). Several of these genes (e.g., cluster I in *L. deliciosus*) are even specific to a single *Lactarius* species. These species-specific clusters are not enriched in MiSSPs, in contrast to other EcM interactions (Kohler et al., 2015; Miyauchi et al., 2020).

## Discussion

Ectomycorrhizal fungi in Russulaceae, including *Lactarius* and *Russula* species, are prominent symbionts in many boreal and temperate forest ecosystems (Looney et al., 2018). However, the mechanisms underlying their symbiosis development and functioning, and the expression of their functional traits in natural settings are unknown. Owing to on-going large-scale genomic initiatives, we can now use unparalleled genomic resources to reconstruct the evolutionary history of saprotrophic and mycorrhizal fungi inhabiting forests and woodlands, and the genomic patterns that underlie the diversity of fungal modes of nutrition (Kohler et al., 2015; Miyauchi et al., 2020). In addition, individual reference genomes can be used to study species-specific traits. Identifying whether different lineages of *Lactarius* species having contrasted biology and ecology differ in their symbiosis-related gene repertoires is one of the main goals of the present study. We thus compared the symbiotic gene repertoires of four *Lactarius* species in association with different pine hosts.

### Transcript Profiling of *L. deliciosus–P. taeda* Ectomycorrhiza Development

During the development of the *L. deliciosus–P. taeda* association, the initiation stage is marked by a profuse attachment of hyphae onto short roots and the formation of a limited number of ectomycorrhizal root tips. At this early contact stage, only six fungal genes were significantly regulated. This gene expression pattern contrasts with the massive transcriptional regulation, involving hundreds of genes, taking place upon attachment of *L. bicolor* and *Hebeloma cylindrosporum* hyphae on short roots (Plett et al., 2015; Doré et al., 2017). This may indicate that the mechanisms involved in host root recognition and early root colonization strikingly differ between fungi forming EcM symbioses. Among the six early symbiosis-regulated genes, two genes coding for small cysteine-rich CPPs are repressed. Fungal CPPs form ordered aggregates on plant cell walls and act as microbe/pathogen-associated molecular patterns (MAMPs/PAMPs) and virulence factors (Gaderer et al., 2014; Pazzagli et al., 2014; Baccelli, 2015), suggesting that the down-regulation of CCP genes dampens the induction of plant defense responses when *L. deliciosus* hyphae attached to roots. Interestingly, a strong down-regulation of a CCP gene was also reported in the Ascomycota *Tuber melanosporum* upon symbiosis (Martin et al., 2010). Recently, some CPPs were further found in several other Basidiomycota EcM fungi (Pellegrin et al., 2015). This consistency of regulation and distribution may suggest a conserved role of CPPs in EcM symbioses. The expression of a carboxylic acid membrane transporter gene is up-regulated during this earlier stage. Carboxylic acids have been found in root exudates (Neumann and Römheld, 1999; Dakora and Phillips, 2002), the up-regulation of their transporter gene likely facilitates the use of these carbon compounds before being able to acquire soluble carbohydrates released into root apoplastic space.

After their initial attachment, *L. deliciosus* hyphae colonize the apoplastic space and differentiate the Hartig net. In several ectomycorrhizal associations, the root penetration and differentiation of the symbiotic interface are accompanied by a remodeling of both the fungal and plant cell walls (Balestrini and Bonfante, 2014), possibly involving carbohydrate-acting enzymes, such as the GH5-CBM1 endoglucanase (Zhang et al., 2018). Indeed, the two up-regulated genes coding for xyloglucan-specific endo-β-1,4-glucanases (GH45) may be involved in the plant cell wall remodeling during the construction of the Hartig net, while the chitin deacetylase (CE4) could facilitate the hyphal penetration via deacetylation which protects chitin from being hydrolyzed by plant chitinases and thus evades plant immunity. Of note, no pectin targeting CAZyme gene was found to be significantly regulated in *L. deliciosus*, although an up-regulation of endo-/exo-(rhamno)galacturonase family (GH28) genes has been reported in both *L. bicolor* and *T. melanosporum* EcM (Martin et al., 2008, 2010). Intriguingly, no gene coding for GH28 was found in *Lactarius* genomes (Lebreton et al., in preparation). The lack of GH28 gene in *Lactarius* genomes suggests that root colonization by *L. deliciosus* does not involve pectin degradation.

The penetration into the root apoplastic space also requires the attenuation of host defenses involving effector-like MiSSP genes (Plett et al., 2011, 2014, 2020; Kang et al., 2020). Compared to *L. bicolor, P. microcarpus*, and *H. cylindrosporum*, only a few MiSSPs and MrSSPs (i.e., 38 transcripts) have been detected during pine root colonization by *L. deliciosus*. As expected, none showed a sequence similarity to the known MiSPPs, such as LbMiSSP7, LbMiSSP7.6, LbMiSSP8, or PaMiSSP10b (Plett et al., 2011, 2014, 2020; Kang et al., 2020).

More than 20 membrane transporter/permease genes are induced in the middle and late developmental stages of *L. deliciosus–P. taeda* ectomycorrhizas, in agreement with the reported induction of a wide array of nutrient transporters in other mycorrhizal assocations (Garcia et al., 2016). Notably, the number of induced genes encoding oligopeptide and carboxylic acid transporters is higher than the set of sugar and amino acid transporters, suggesting that oligopeptides and carboxylic acids are the major N and C sources for *Lactarius* spp. during its symbiosis with *Pinus*. Organic N, such as amino acids and oligopeptides, were proposed as the major forms transferred to the host in orchid mycorrhizas, based on the large induction of corresponding transporters especially in the symbiotic protocorms (Fochi et al., 2017; Valadares et al., 2021). Unlike orchid mycorrhizal fungi, EcM fungi, however, are known to possess only a limited saprotrophic ability (Lindahl and Tunlid, 2015). Given the lack of organic N in the growth medium used in our EcM assay, the organic N transporters induced in *L. deliciosus* are likely involved in N transfer at the plant-fungus interface, rather than N uptake from the growth medium. The organic N pool at the symbiotic interface could result from the intensive cleavage of root apoplastic and/or cell wall proteins by the large set of secreted proteases whose genes are concomitantly induced in *L. deliciosus*. Several TGL genes are induced and their increased expression likely support the massive formation of the membrane-rich finger-like hyphal structures of the symbiotic interface. The observed changes in gene expression are likely under the control of transcriptional regulators (Daguerre et al., 2017). Only part of the identified transcription factor genes were up-regulated in both the middle and late developmental stages.

Notably, a large number of DEGs were predicted to code for proteins of unknown functions, especially among the repressed genes. The functional characterization of the most highly regulated stage-specific DEGs of unknow function, will provide a better understanding of their role in symbiosis development. Our time-course transcript profiling of the *L. deliciosus–P. taeda* association showed that only a few genes are induced during the earlier colonization stage, then a substantial transcriptional reprogrammation is taking place during the formation of the Hartig net and mantle sheath. Transcriptome changes mainly concern genes involved in nutrient uptake and primary metabolism.

### Secreted Proteases Are Important Elements in the Symbiotic Toolkit of *Lactarius*

Recent findings have evidenced that proteases secreted by both the fungal pathogen and the host plant are important factors for early partner interactions taking place in the apoplast (Jashni et al., 2015b; Wang et al., 2020). On the one hand, several secreted fungalysins have been reported to enhance the pathogen invasion by cleaving plant secreted chitinases (Naumann et al., 2011; Jashni et al., 2015a; Sanz-Martín et al., 2016; Ökmen et al., 2018). On the other hand, the host plant can also release proteases to inhibit the growth and invasion of pathogens through direct cleavage of their cell wall protein components or triggering plant defense via cleavage of their secreted effector proteins (Jashni et al., 2015b; Wang et al., 2020, 2021). During the middle and late stages of *L. deliciosus–P. taeda* EcM development, 81 transcripts coding for secreted proteases accumulated at a much higher level in colonizing *L. deliciosus* hyphae. This set of symbiosis-related protease genes accounts for 80% of the total secreted protease gene repertoire in *L. deliciosus* genome. This striking induction of protease genes during root penetration strongly suggests their involvement in the colonization. In contrast, only a few genes coding for secreted proteases are up-regulated in *L. bicolor* interacting with poplar and Douglas fir hosts, while most of them being down-regulated upon symbiosis (Plett et al., 2015). In ericoid mycorrhizal fungi, 6–21% of genes coding for secreted proteases are up-regulated during symbiosis (Martino et al., 2018). Similarly, a few protease genes in orchid mycorrhizal fungi were also found to be regulated during symbiosis (Balestrini et al., 2014; Valadares et al., 2021).

We found several fungalysin genes induced during *L. deliciosus* symbiosis, and almost all of these proteases are evolutionarily conserved in multiple fungi including pathogens (Figure 3). Interestingly, an expansion of fungalysin genes has been reported in several other symbiotic fungi, such as *L. bicolor*, the orchid mycorrhizal *Tulasnella calospora* and the endophytic *Piriformospora indica*, and some members were also up-regulated when interacting with plant hosts (Zuccaro et al., 2011, 2014; Balestrini et al., 2014). This conservation of sequence and regulation suggests that these secreted fungalysins could facilitate the root colonization via targeting and cleaving defense-related host proteins or enzymes in the symbiotic interface. We also found an unusual enrichment of sedolisins (S53) in the EcM-induced genes. This appears to be unique to *Lactarius* so far, as massive up-regulation of sedolisin genes has not been reported in any other ectomycorrhizal fungi (Kohler et al., 2015; Miyauchi et al., 2020). Although a regulation of some sedolisin genes was observed in a few plant-interacting fungi, such as the necrotrophic pathogen *Botrytis cinerea*, their role(s) in the interaction remain largely unknown (Choquer et al., 2021). However, their dramatic up-regulation observed in the present study and the striking expansion in *Lactarius* genomes (Looney et al., 2021; Lebreton et al., in preparation), both suggest their involvement in *Lactarius* symbiosis. Since RNAs used for transcriptomic profilings were extracted from whole ectomycorrhizal tips/clusters, we could not preclude the possibility that some of these proteases are secreted by extraradical hyphae to scavenge nitrogen present in the mycorrhizosphere. Nonetheless, given the absence of organic N in the growth medium used in our *in vitro* system and the rare presence of extraradical hyphae during root penetration (Figure 1E), we postulate that most, if not all, of the induced secreted proteases are acting in the symbiotic interface. Further tissue-specific transcriptomics, proteomics or protein immunolocalization studies should provide additional information on their subcellular locations and function(s).

### Highly Specific Symbiotic Gene Repertoires Encoded by *Lactarius*: Possible Links to the Host-Specificity

To better understand the contrasting specificity of *Lactarius* symbionts (i.e., different fungi specific to different pines), we investigated the transcriptional basis of this difference by comparing the symbiotic gene repertoires of four *Lactarius* species, each colonizing a compatible host. Surprisingly, a very low number of orthologous genes (belonging to 24 OGs) were found to be commonly up-regulated in all four *Lactarius* species, whereas most of OGs had a regulation specific to a single species (Supplementary Figure 4). This finding suggests that each *Lactarius* species expresses a highly specific symbiotic gene set to interact with its specific host(s). Interestingly, this specificity of gene regulation seems to be more related to the fungus identity and less to the host pine because the two fungi (*L. deliciosus* and *L. vividus*) colonizing the same host pine species (*P. taeda*) under our conditions, have no more commonly regulated OGs. However, to better understand how *Lactarius* gene regulation is influenced by host identity further comparison of interactions between the same fungus and different hosts is required. The high specificity of the gene regulation in *Lactarius–Pinus* EcM contrasts with the results reported on *Suillus–Pinus* EcM associations in which a majority of *Suillus* species transcripts (corresponding to 231 orthologous genes/OGs) are similarly up-regulated in response to different compatible host pine species (Liao et al., 2016). Even with a possibility of overestimation due to the *de novo* meta-transcriptomic strategy (Liao et al., 2016), the observed difference may reflect the occurrence of specific genetic factors to control host-specificity in taxonomically divergent fungal orders (Russulales vs. Boletales).

Expectedly, the genes commonly up-regulated in all *Lactarius* species are mainly involved in the core processes of the symbiosis functioning, such as the nutrient transport and plant cell wall modification. The presence of several secreted protease genes further supports their essential role in *Lactarius* symbioses. Importantly, we found in the specifically up-regulated OG sets, several genes whose homologs in other fungi could function as host-specificity determinants. For instance, the fungalysin UmFly1 secreted by the pathogenic fungus *Ustilago maydis* cleaves its host chitinase allowing a successful infection, while its ortholog from a close, but non-pathogenic relative could not (Ökmen et al., 2018). Lectins were the first characterized molecules which played a decisive role in *L. deterrimus-*spruce specificity (Guillot et al., 1991; Giollant et al., 1993). In addition, MiSSPs could be key factors during specific interactions due to their high sequence specificity as observed in many EcM fungi (Kohler et al., 2015; Miyauchi et al., 2020). Finally, some of the species-specific genes (Figure 5 and Supplementary Figures 5–7) could also play a role in the host-specificity of *Lactarius* spp. although their function is yet unknown.

In brief, the present transcriptome profiling of *Lactarius* ectomycorrhizas are providing new insights on the genetic basis of symbiosis development in an ecologically important ectomycorrhizal order, the Russulales, which was not investigated so far. We highlighted several unique symbiosis-related features which have not been observed in other ectomycorrhizal lineages. This includes a striking induction of dozens of secreted protease genes, suggesting that a protease-based crosstalk occurrs between *Lactarius* species and their host(s) during ectomycorrhizal development. As found in the plant pathogenic smut, *Ustilago maydis* (Ökmen et al., 2018), secreted proteases may function as a new class of symbiosis-related effectors and host-specificity factors in *Lactarius* species. Unlike *Suillus* species, *Lactarius* symbionts express highly specific symbiotic gene sets when interacting with their specific hosts. It remains to determine which symbiosis-regulated conserved genes and species-specific genes play a role in their host-specificity.

## Data Availability Statement

The original contributions presented in the study are publicly available. All clean reads were submitted to National Center for Biotechnology Information (NCBI) Sequence Read Archive (SRA) under the BioProject of PRJNA706172.

## Author Contributions

FY, NT, and FM designed the project. NT performed the biological experiments with assistance from WX. AL analyzed the transcriptome data. NT, AL, and FM interpreted the data and wrote the manuscript with the help of FY and YD. All authors approved the final manuscript.

## Conflict of Interest

The authors declare that the research was conducted in the absence of any commercial or financial relationships that could be construed as a potential conflict of interest.

## Publisher’s Note

All claims expressed in this article are solely those of the authors and do not necessarily represent those of their affiliated organizations, or those of the publisher, the editors and the reviewers. Any product that may be evaluated in this article, or claim that may be made by its manufacturer, is not guaranteed or endorsed by the publisher.

## Footnotes

1
https://mycocosm.jgi.doe.gov/mycocosm

2
https://github.com/TransDecoder/TransDecoder

3
https://genome.jgi.doe.gov/portal/

## Supplementary Material

Supplementary Figure 1 *In vitro* pouch system for EcM synthesis **(A)** and major steps involved **(B)**.

Supplementary Figure 2 Mycorrhization assay of *Lactarius.* Each of the four *Lactarius* species was paired with five different *Pinus* species to assay their ectomycorrhizal compatibility **(A)**; Compatible *Lactarius–Pinus* combinations leading to symbiosis development are indicated as “+” while incompatible combinations as “–”. The four interactions highlighted in orange were used for RNA sequencing. In addition to the *L. deliciosus–P. taeda* ectomycorrhizal roots shown in Figure 1, ectomycorrhizal roots of three other compatible combinations were checked by stereomicroscopy, including *L. akahatsu* with *P. tabuliformis* **(B)**, *L. sanguifluus* with *P. sylvestris* **(C)**, and *L. vividus* with *P. taeda* **(D)**. Agarose-embedded *L. vividus–P. taeda* sections (50 μm), stained with WGA-FITC were further checked for the presence of mantle and Hartig net **(E)**. Abbreviations: M, Mantle; Hn, Hartig net; Pm, *Pinus massoniana*; Ps, *P. sylvestris*; Ptab, *P. tabuliformis*; Ptae, *P. taeda*; and Py, *P. yunnanensis*. Scale bar in **(B–D)**: 1 mm and in **(E)**: 100 μm.

Supplementary Figure 3 Venn diagram showing the number of commonly and specifically induced *L. deliciosus* genes between 4 and 6 wpi. Based on the functional annotation, these induced genes were classified into nine categories including proteases, CAZymes, transporters, transcription factors, SSPs, cytochrome p450, lipases, lectins and unknown.

Supplementary Figure 4 Orthogroups (OGs) containing commonly and specifically regulated genes among four *Lactarius species*. Abbreviations: Lacaka, *L. akahatsu*; Lacdel, *L. deliciosus*; Lacsan, *L. sanguifluus*; and Lacviv, *L.vividus*.

Supplementary Figure 5 Phylogenetic conservation of *L. akahatsu* differentially expressed genes (DEGs). BLASTp identity heatmap and function classification barplot were generated with the same method described in Figure 5.

Supplementary Figure 6 Phylogenetic conservation of *L. sanguifluus* differentially expressed genes (DEGs). BLASTp identity heatmap and function classification barplot were generated with the same method described in Figure 5.

Supplementary Figure 7 Phylogenetic conservation of *L. vividus* differentially expressed genes (DEGs). BLASTp identity heatmap and function classification barplot were generated with the same method described in Figure 5.

Supplementary Table 1 Details of the fungal samples used for RNA sequencing and quality of reads. Wpi, weeks post inoculation; FLM, free-living mycelia.

Supplementary Table 2 Newly predicted *Lactarius* genes and their functionnal annotations.

Supplementary Table 3 *Lactarius* genes induced during Mycorrhization. Data are split in different sheets according to the species: *L. akahatsu* (Sheet S3A), *L. deliciosus* (Sheet S3B), *L. sanguifluus* (Sheet S3C), and *L. vividus* (Sheet S3D); gene functional annotation (secretion, lipase, CAZyme, lectin, subtilase, EC number, PFAM domain, KOG, cluster ID, and orhogroup ID) and expression (TPM) in each sample are also included in this table.

Supplementary Table 4 *Lactarius* genes repressed during Mycorrhization. Data are split in different sheets according to the species: *L. akahatsu* (Sheet S4A), *L. deliciosus* (Sheet S4B), *L. sanguifluus* (Sheet S4C), and *L. vividus* (Sheet S4D); gene functional annotation (secretion, lipase, CAZyme, lectin, subtilase, EC number, PFAM domain, KOG, cluster ID, and orhogroup ID) and expression (TPM) in each sample are also included in this table.
