# Supplementary figures and images for: Transcriptome Profiling Reveals Differential Gene Expression of Secreted Proteases and Highly Specific Gene Repertoires Involved in Lactarius–Pinus Symbioses
Source: Front Plant Sci. 2021 Aug 19;12:714393. doi: 10.3389/fpls.2021.714393 (PMC8417538; doi:10.3389/fpls.2021.714393)

## Slide 1
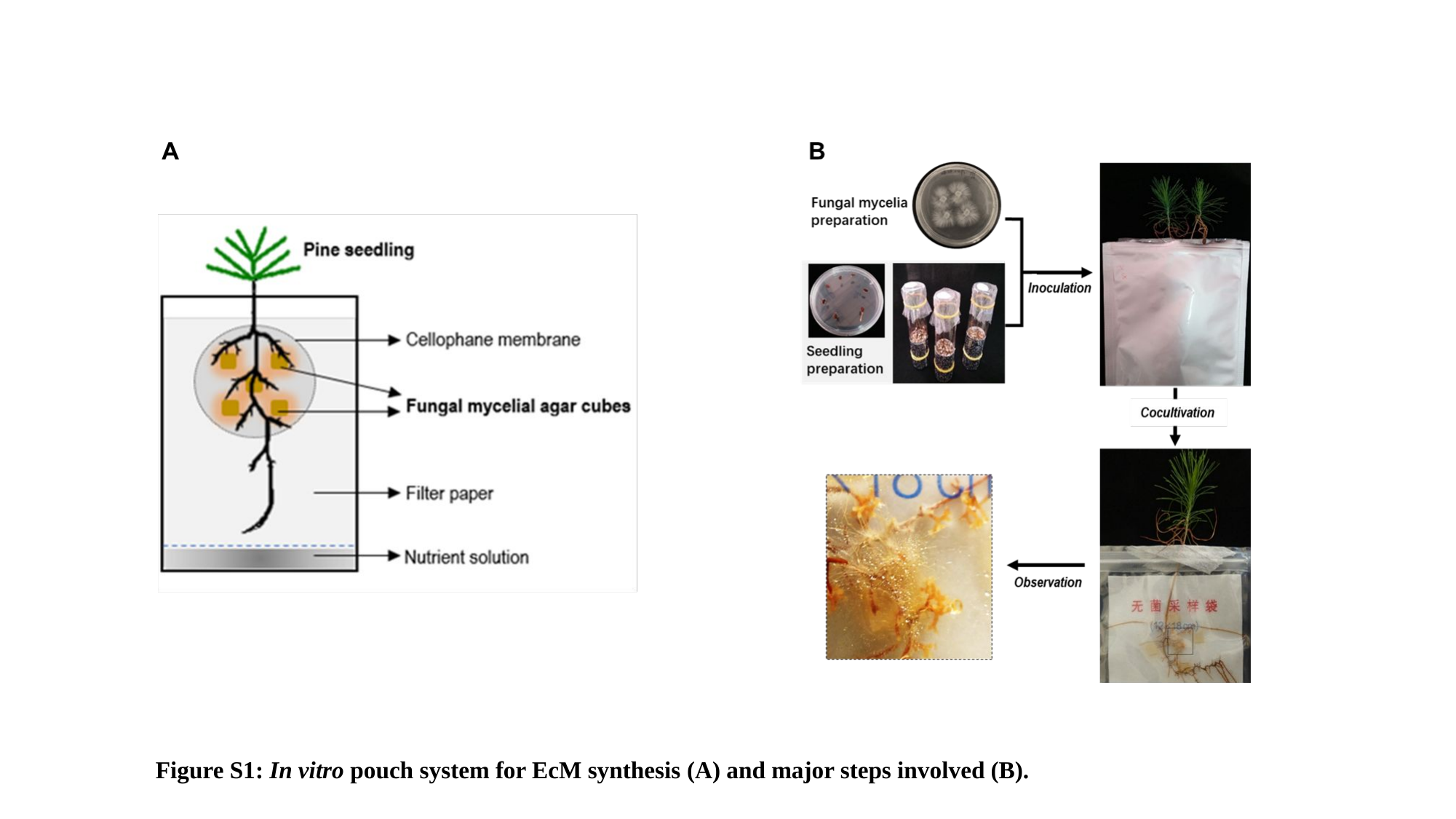

Figure S1: In vitro pouch system for EcM synthesis (A) and major steps involved (B).

Supplement: Supplementary Figure 1 — In vitro pouch system for EcM synthesis (A) and major steps involved (B). [file Presentation_1.PPTX]
