# Supplementary material for: Transcriptome Profiling Reveals Differential Gene Expression of Secreted Proteases and Highly Specific Gene Repertoires Involved in Lactarius–Pinus Symbioses
Source: Front Plant Sci. 2021 Aug 19;12:714393. doi: 10.3389/fpls.2021.714393 (PMC8417538; doi:10.3389/fpls.2021.714393)
Supplement: Supplementary Figure 2 — Mycorrhization assay of Lactarius. Each of the four Lactarius species was paired with five different Pinus species to assay their ectomycorrhizal compatibility (A); Compatible Lactarius–Pinus combinations leading to symbiosis development are indicated as “+” while incompatible combinations as “–”. The four interactions highlighted in orange were used for RNA sequencing. In addition to the L. deliciosus–P. taeda ectomycorrhizal roots shown in Figure 1, ectomycorrhizal roots of three other compatible combinations were checked by stereomicroscopy, including L. akahatsu with P. tabuliformis (B), L. sanguifluus with P. sylvestris (C), and L. vividus with P. taeda (D). Agarose-embedded L. vividus–P. taeda sections (50 μm), stained with WGA-FITC were further checked for the presence of mantle and Hartig net (E). Abbreviations: M, Mantle; Hn, Hartig net; Pm, Pinus massoniana; Ps, P. sylvestris; Ptab, P. tabuliformis; Ptae, P. taeda; and Py, P. yunnanensis. Scale bar in (B–D): 1 mm and in (E): 100 μm. [file Presentation_2.PPTX]

## Slide 1
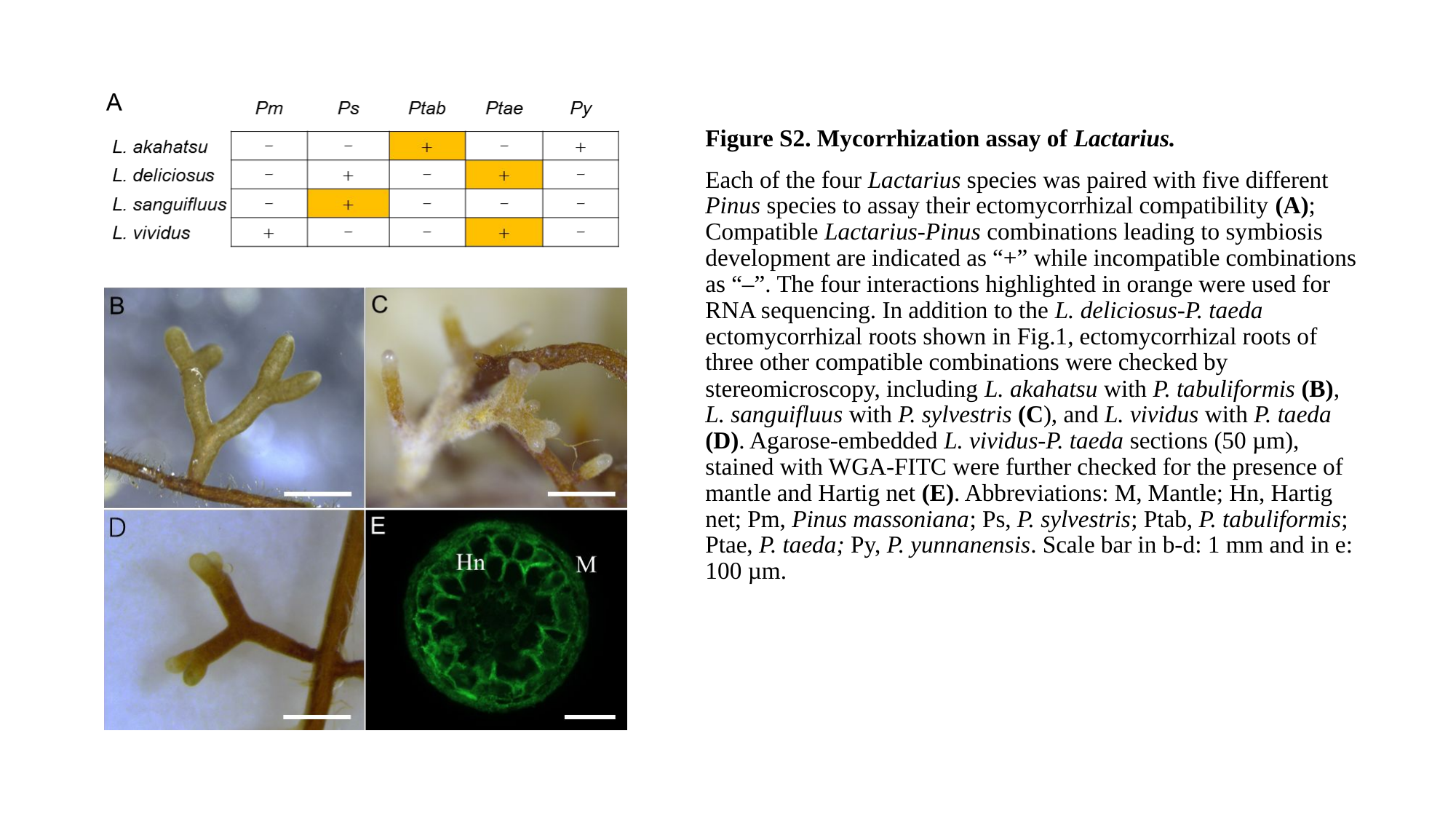

Figure S2. Mycorrhization assay of Lactarius.
Each of the four Lactarius species was paired with five different Pinus species to assay their ectomycorrhizal compatibility (A); Compatible Lactarius-Pinus combinations leading to symbiosis development are indicated as “+” while incompatible combinations as “–”. The four interactions highlighted in orange were used for RNA sequencing. In addition to the L. deliciosus-P. taeda ectomycorrhizal roots shown in Fig.1, ectomycorrhizal roots of three other compatible combinations were checked by stereomicroscopy, including L. akahatsu with P. tabuliformis (B), L. sanguifluus with P. sylvestris (C), and L. vividus with P. taeda (D). Agarose-embedded L. vividus-P. taeda sections (50 µm), stained with WGA-FITC were further checked for the presence of mantle and Hartig net (E). Abbreviations: M, Mantle; Hn, Hartig net; Pm, Pinus massoniana; Ps, P. sylvestris; Ptab, P. tabuliformis; Ptae, P. taeda; Py, P. yunnanensis. Scale bar in b-d: 1 mm and in e: 100 µm.
