# Supplementary material for: Transcriptome Profiling Reveals Differential Gene Expression of Secreted Proteases and Highly Specific Gene Repertoires Involved in Lactarius–Pinus Symbioses
Source: Front Plant Sci. 2021 Aug 19;12:714393. doi: 10.3389/fpls.2021.714393 (PMC8417538; doi:10.3389/fpls.2021.714393)
Supplement: Supplementary Figure 3 — Venn diagram showing the number of commonly and specifically induced L. deliciosus genes between 4 and 6 wpi. Based on the functional annotation, these induced genes were classified into nine categories including proteases, CAZymes, transporters, transcription factors, SSPs, cytochrome p450, lipases, lectins and unknown. [file Presentation_3.PPTX]

## Slide 1
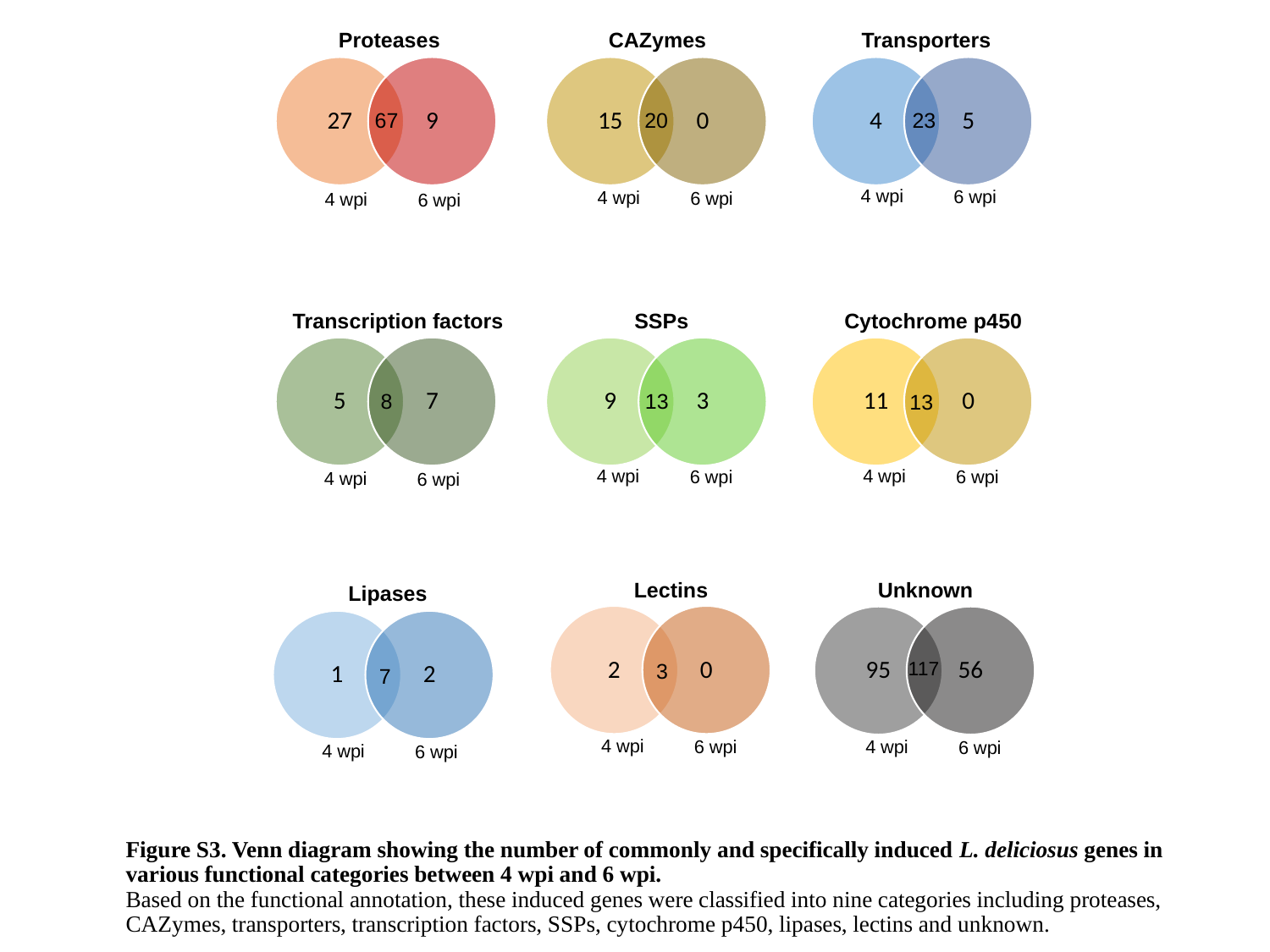

Proteases
CAZymes
Transporters
23
20
67
4 wpi
6 wpi
4 wpi
6 wpi
4 wpi
6 wpi
Transcription factors
SSPs
Cytochrome p450
8
13
13
4 wpi
4 wpi
6 wpi
6 wpi
4 wpi
6 wpi
Lectins
Unknown
Lipases
 117
 3
7
4 wpi
4 wpi
6 wpi
6 wpi
4 wpi
6 wpi
# Figure S3. Venn diagram showing the number of commonly and specifically induced L. deliciosus genes in various functional categories between 4 wpi and 6 wpi. Based on the functional annotation, these induced genes were classified into nine categories including proteases, CAZymes, transporters, transcription factors, SSPs, cytochrome p450, lipases, lectins and unknown.
