# Supplementary material for: Transcriptome Profiling Reveals Differential Gene Expression of Secreted Proteases and Highly Specific Gene Repertoires Involved in Lactarius–Pinus Symbioses
Source: Front Plant Sci. 2021 Aug 19;12:714393. doi: 10.3389/fpls.2021.714393 (PMC8417538; doi:10.3389/fpls.2021.714393)
Supplement: Supplementary Figure 4 — Orthogroups (OGs) containing commonly and specifically regulated genes among four Lactarius species. Abbreviations: Lacaka, L. akahatsu; Lacdel, L. deliciosus; Lacsan, L. sanguifluus; and Lacviv, L.vividus. [file Presentation_4.PPTX]

## Slide 1
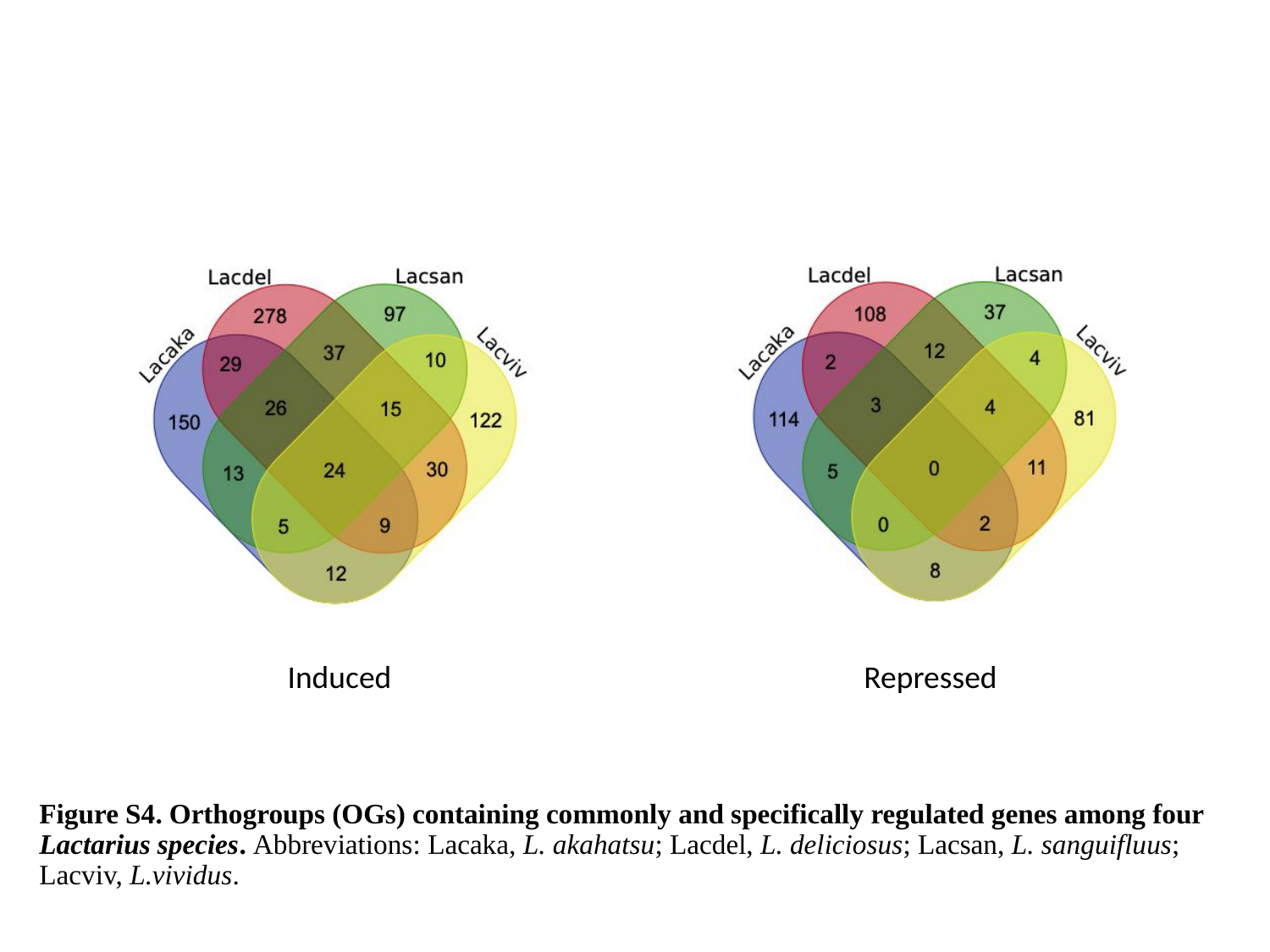

Induced
Repressed
# Figure S4. Orthogroups (OGs) containing commonly and specifically regulated genes among four Lactarius species. Abbreviations: Lacaka, L. akahatsu; Lacdel, L. deliciosus; Lacsan, L. sanguifluus; Lacviv, L.vividus.
