# Supplementary material for: Transcriptome Profiling Reveals Differential Gene Expression of Secreted Proteases and Highly Specific Gene Repertoires Involved in Lactarius–Pinus Symbioses
Source: Front Plant Sci. 2021 Aug 19;12:714393. doi: 10.3389/fpls.2021.714393 (PMC8417538; doi:10.3389/fpls.2021.714393)
Supplement: Supplementary Figure 7 — Phylogenetic conservation of L. vividus differentially expressed genes (DEGs). BLASTp identity heatmap and function classification barplot were generated with the same method described in Figure 5. [file Data_Sheet_3.PDF]

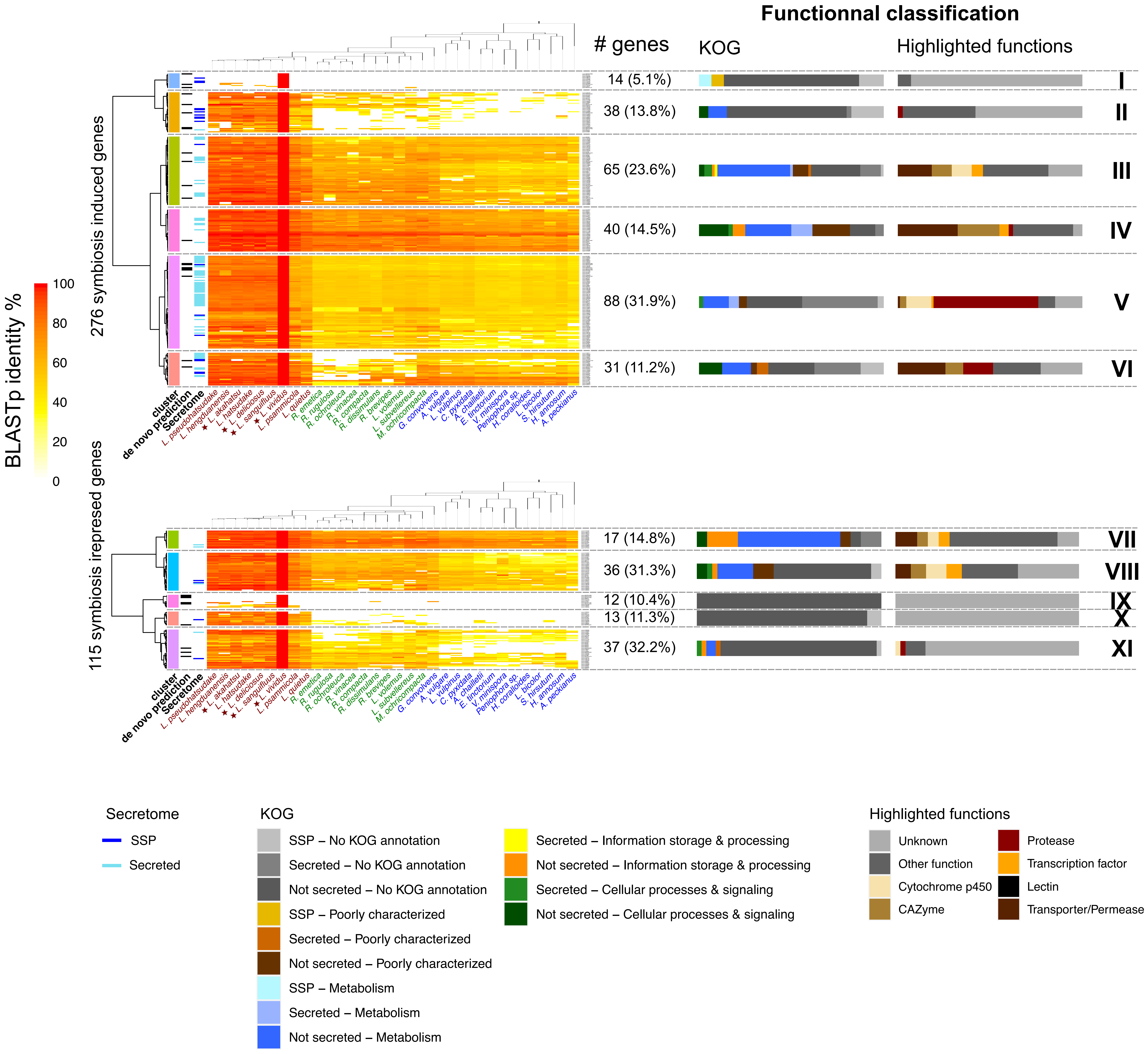
Functionnal classification
# genes
KOG
Highlighted functions
276 symbiosis induced genes
115 symbiosis irepresed genes
BLASTp identity %
100
80
60
40
20
0
14 (5.1%)
I
38 (13.8%)
II
65 (23.6%)
III
40 (14.5%)
IV
88 (31.9%)
V
31 (11.2%)
VI
17 (14.8%)
VII
36 (31.3%)
VIII
12 (10.4%)
IX
13 (11.3%)
X
37 (32.2%)
XI
cluster
de novo prediction
Secretome
L. pseudohatsudake
L. hengduanensis
L. akahatsu
L. hatsudake
L. deliciosus
L. sanguifluus
L. vividus
L. psammicola
L. quietus
R. emetica
R. rugulosa
R. ochroleuca
R. vinacea
R. compacta
R. dissimulans
R. brevipes
L. volemus
L. subvellereus
M. ochricompacta
G. convolvens
A. vulgare
L. vulpinus
C. pyxidata
A. chailletii
E. tinctorium
V. minispora
Peniophora sp.
H. coralloides
L. bicolor
S. hirsutum
H. annosum
A. peckianus
Secretome
SSP
Secreted
KOG
SSP – No KOG annotation
Secreted – No KOG annotation
Not secreted – No KOG annotation
SSP – Poorly characterized
Secreted – Poorly characterized
Not secreted – Poorly characterized
SSP – Metabolism
Secreted – Metabolism
Not secreted – Metabolism
Secreted – Information storage & processing
Not secreted – Information storage & processing
Secreted – Cellular processes & signaling
Not secreted – Cellular processes & signaling
Highlighted functions
Unknown
Other function
Cytochrome p450
CAZyme
Protease
Transcription factor
Lectin
Transporter/Permease
