# Supplementary material for: Transcriptome Profiling Reveals Differential Gene Expression of Secreted Proteases and Highly Specific Gene Repertoires Involved in Lactarius–Pinus Symbioses
Source: Front Plant Sci. 2021 Aug 19;12:714393. doi: 10.3389/fpls.2021.714393 (PMC8417538; doi:10.3389/fpls.2021.714393)
Supplement: Supplementary Figure 5 — Phylogenetic conservation of L. akahatsu differentially expressed genes (DEGs). BLASTp identity heatmap and function classification barplot were generated with the same method described in Figure 5. [file Data_Sheet_1.PDF]

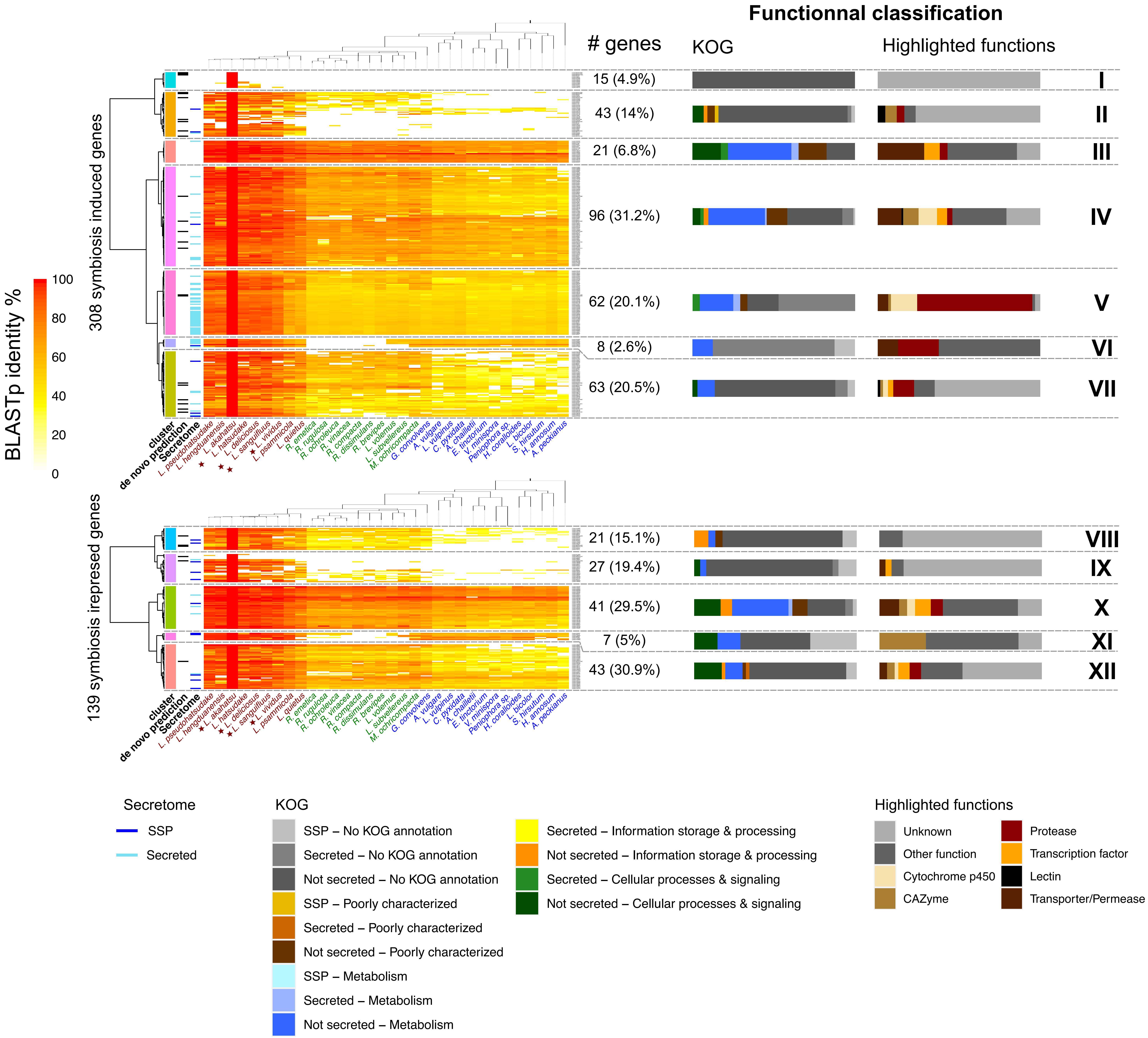

Functionnal classification
genes
KOG
Highlighted functions
308 symbiosis induced genes
139 symbiosis irepresed genes
BLASTp identity %
100
80
60
40
20
0
15 (4.9%)
I
43 (14%)
II
21 (6.8%)
III
96 (31.2%)
IV
62 (20.1%)
V
8 (2.6%)
VI
63 (20.5%)
VII
21 (15.1%)
VIII
27 (19.4%)
IX
41 (29.5%)
X
7 (5%)
XI
43 (30.9%)
XII
cluster
de novo prediction
Secretome
L. pseudohatsudake
L. hengduanensis
L. akahatsu
L. hatsudake
L. deliciosus
L. sanguifluus
L. vividus
L. psammicola
L. quietus
R. emetica
R. rugulosa
R. ochroleuca
R. vinacea
R. compacta
R. dissimulans
R. brevipes
L. volemus
L. subvellereus
M. ochricompacta
G. convolvens
A. vulgare
L. vulpinus
C. pyxidata
A. chailletii
E. tinctorium
V. minispora
Peniophora sp.
H. coralloides
L. bicolor
S. hirsutum
H. annosum
A. peckianus
Secretome
SSP
Secreted
KOG
SSP – No KOG annotation
Secreted – No KOG annotation
Not secreted – No KOG annotation
SSP – Poorly characterized
Secreted – Poorly characterized
Not secreted – Poorly characterized
SSP – Metabolism
Secreted – Metabolism
Not secreted – Metabolism
Secreted – Information storage & processing
Not secreted – Information storage & processing
Secreted – Cellular processes & signaling
Not secreted – Cellular processes & signaling
Highlighted functions
Unknown
Other function
Cytochrome p450
CAZyme
Protease
Transcription factor
Lectin
Transporter/Permease
